# Microfluidic Paper-Based Devices

**DOI:** 10.3390/mi16030307

**Published:** 2025-03-06

**Authors:** Lung-Ming Fu

**Affiliations:** Department of Engineering Science, National Cheng Kung University, Tainan 701, Taiwan; loudyfu@mail.ncku.edu.tw

Since their development by Whitesides’ group in 2007 [1], microfluidic paper-based analytical devices (μPADs) have emerged as a transformative technology in point-of-care (POC) diagnostics, food safety, environmental monitoring, and clinical toxicology. These μPADs leverage the capillary action of paper to transport liquids without external pumps or power sources. This, together with their low cost, portability, and ease of use, renders μPADs of great interest for the rapid and reliable detection of analytes in resource-limited settings.

Over the past decade, researchers have continued to improve fabrication techniques and detection mechanisms to further enhance the accuracy, sensitivity, reproducibility, applicability, durability, and multiplexing capabilities of μPADs. Various fabrication techniques, including wax printing, screen printing, and laser etching, have been explored to optimize the fluid flow and form well-defined reaction zones [2]. In addition, various polymeric and hybrid paper-based substrates have been proposed to enhance the mechanical strength and chemical resistance of lab-on-a-chip (LoC) devices [3]. Such devices are typically made from cellulose paper, chemically modified to create fluidic pathways, and allow the integration of various detection methodologies, including colorimetric, electrochemical, fluorescence, and distance-based detection [4,5].

One of the most impactful applications of μPADs is POC diagnostics, where they have proven to be extremely effective in detecting infectious diseases such as malaria and HIV [6]. They have also been successfully used in more advanced applications, such as early cancer biomarker detection, thus, offering the potential to revolutionize cancer diagnostics by providing non-invasive, accessible, and cost-effective solutions [7]. Rapid electrolyte monitoring is critical in many clinical settings, particularly for patients with electrolyte imbalances. To address this need, Lu et al. [8] presented a microfluidic fluorescence detection platform for whole blood sodium (Na^+^) determination. Their work highlighted the precision and speed of paper-based systems for electrolyte assessment. Burgos-Flórez et al. [9] developed a μPAD for blood plasma separation and biomarker detection, further demonstrating the potential of paper-based devices for early disease diagnosis. Since the COVID-19 pandemic, μPADs have played a crucial role in enabling the rapid detection of viral infection through isothermal nucleic acid amplification tests [2,10].

The COVID-19 pandemic demonstrated the urgent need for rapid, decentralized diagnostic tools capable of detecting viral infections at a low cost and with minimal medical experience [10]. In addition to their role in detecting the COVID-19 virus and its subsequent variants, µPADs have been developed to detect SARS-CoV-2 antigens and nucleic acids using colorimetric and electrochemical methods. These devices offer many advantages over traditional benchtop instruments, including faster turnaround times, minimal sample preparation, and the ability to operate without a sophisticated laboratory infrastructure. Consequently, µPADs are expected to undergo intense research to provide even faster and more effective responses in possible viral outbreaks in the future.

Besides public health, food safety is also a major global concern, as contaminants, preservatives, and pathogens can pose substantial risks to human health. Traditional food safety testing methods are often labor intensive and require sophisticated laboratory equipment. However, µPADs have demonstrated significant potential for revolutionizing food safety analysis by offering rapid, cost-effective, and user-friendly alternatives [3]. For instance, Chen et al. [11] presented a rapid µPAD system for the fast, accurate, and portable detection of sodium dehydroacetate in food samples. Ko et al. [12] developed a finger-pump-based microfluidic detection system for the detection of methylparaben preservative in foods [12]. Ireta-Muñoz et al. [13] proposed a non-invasive paper-based approach for the low-cost evaluation of milk quality in the dairy industry. Zhang et al. [14] presented a µPAD with an embedded immobilized enzyme microreactor zone for the detection of organophosphorus pesticide residues in food samples [14]. These diverse applications of μPADs in food safety demonstrate the versatility and effectiveness of μPAD technology in enabling rapid, accessible, and reliable food safety analyses across various sectors of the food industry.

Heavy metal pollution poses serious risks to the environment and health, necessitating the development of rapid and efficient detection methods. Microfluidic paper-based devices have demonstrated immense potential for the detection of heavy metals in environmental and water samples. For example, Silva et al. [15] developed a nonequilibrium potentiometric sensor integrated with a metal-modified paper-based substrate to measure lead (Pb^2+^) concentrations in simulated environmental samples. Yuan et al. [16] presented a portable multi-channel fluorescence µPAD to simultaneously detect four heavy metals (Pb^2+^, Hg^2+^, Cd^2+^, and As^3+^) in apple and lettuce samples using smartphone imaging. Al-Jaf et al. [17] proposed a µPAD impregnated with fluorescent MOF@tetracycline nanocomposite for the simultaneous detection of copper (Cu^2+^) and iron (Fe^3^+) ions in drinking water using a simple colorimetric method [17]. Behbahan et al. [18] concluded that µPADs combined with metal–organic frameworks (MOFs) provide enhanced selectivity, sensitivity, and detection efficiency for heavy metal detection. These advancements in μPAD technology for heavy metal detection illustrate its ability to address complex environmental contamination issues using portable, sensitive, and multi-analyte testing platforms.

Microfluidic paper-based sweat sensors have emerged as a viable noninvasive POC technique for monitoring physiological health, reflecting their growing importance in personalized healthcare and remote monitoring applications for evaluating hydration status, stress levels, and overall metabolic function. Yang et al. [19] developed a screen-printed wearable sweat microfluidic paper-based sensor for assessing the hydration status by detecting potassium (K^+^) and sodium (Na^+^) ions. Fiore et al. [20] presented a wearable electrochemical paper-based biosensor for the determination of cortisol in sweat to support the non-invasive monitoring of stress levels. Deng et al. [21] proposed a skin-interfaced bifluidic paper-based device for real-time quantitative sweat analysis based on integrated microfluidic channels with colorimetric and electrochemical detection mechanisms. The development of these diverse μPAD-based sweat sensors illustrates the rapid progress in wearable, non-invasive tools for continuous health assessment and signals with significant potential for advancements in personalized healthcare management, such as real-time stress monitoring, hydration tracking, and early detection of metabolic imbalances.

µPADs are increasingly being used in forensic and clinical toxicology, offering rapid, cost-effective, and portable solutions for the detection of drugs and toxic substances. The minimal sample volumes of such devices make them highly applicable in forensic investigations and clinical diagnostics [22,23]. Recent advances in µPAD technology have enabled the detection of various psychoactive substances and drugs of abuse. Zaki et al. [24] combined a novel 3D-printed microfluidic paper-based column with liquid chromatography-mass spectrometry (LC-MS) to detect methamphetamine, 7-aminonitrazepam, and morphine-3-glucuronide in just one microliter of plasma. The integration of nanomaterials with µPADs further enhances their analytical performance. For instance, Suleman et al. [25] developed an aptamer sensor combined with two-dimensional nanomaterials for the highly selective electrochemical detection of ketamine with a sensitivity at the 10-ppb level. These advancements in μPAD technology, including the integration of nanomaterials and novel detection methods, are expanding the capabilities of forensic and clinical toxicology by enabling the rapid, accurate, and portable detection of a wide range of illicit substances and drugs.

µPADs have the potential to address many critical challenges in healthcare, food safety, and environmental monitoring. Their low cost, portability, and ease of use render them ideal for POC applications, particularly in resource-limited environments. However, despite significant advancements in µPADs in recent years, particularly in the fields of viral detection, nucleic acid amplification, heavy metal detection, and forensic and clinical toxicology, several important challenges remain. These include the need for improved reagent stability, better sensitivity and specificity, more robust fabrication techniques, and improved integration of µPADs with advanced detection methods. In addition, enhancing the reproducibility of µPADs and developing standardized protocols for their use are essential for improving their commercial scalability. Nonetheless, as research continues to advance, µPADs are poised to play an increasingly important role in improving global health outcomes and ensuring safety across diverse fields. Future developments in μPAD technology are likely to leverage emerging technologies such as artificial intelligence and the Internet of Things, potentially creating smart, connected platforms that can revolutionize diagnostics, monitoring, and analysis across healthcare, environmental science, food safety, and many other sectors.
